# Sleep enhances a spatially mediated generalization of learned values

**DOI:** 10.1101/lm.038828.115

**Published:** 2015-10

**Authors:** Amir-Homayoun Javadi, Anisha Tolat, Hugo J. Spiers

**Affiliations:** Institute of Behavioural Neuroscience, Department of Experimental Psychology, University College London, London WC1H 0AP, United Kingdom

## Abstract

Sleep is thought to play an important role in memory consolidation. Here we tested whether sleep alters the subjective value associated with objects located in spatial clusters that were navigated to in a large-scale virtual town. We found that sleep enhances a generalization of the value of high-value objects to the value of locally clustered objects, resulting in an impaired memory for the value of high-valued objects. Our results are consistent with (a) spatial context helping to bind items together in long-term memory and serve as a basis for generalizing across memories and (b) sleep mediating memory effects on salient/reward-related items.

Sleep appears to play an important role in memory consolidation (Diekelmann and Born 2010; Spiers and Bendor 2014; Walker and Stickgold 2010). This has been demonstrated for both declarative (Marshall and Born 2007; Rasch et al. 2007; Lahl et al. 2008) and nondeclarative memories (Fischer et al. 2002; Ditye et al. 2013). Sleep not only appears to improve memory performance but enhances generalization from overlapping experiences (Lewis and Durrant 2011; Inostroza and Born 2013; Stickgold and Walker 2013). Examples can be found in statistical learning (Durrant et al. 2011), relational memory (Ellenbogen et al. 2007; Lau et al. 2011) and false memory paradigms (Payne et al. 2009).

In addition to memory integration, sleep has also been shown to play a role in strengthening memories based on their significance. For example, sleep preferentially consolidates emotional memories, over neutral memories, within procedural memory (Javadi et al. 2011) and declarative memory tasks (Hu et al. 2006; Payne et al. 2008; for review, see Walker (2010)). Similarly, sleep appears to favor memories with future relevance and importance (Wilhelm et al. 2011; Van Dongen et al. 2012; Perogamvros et al. 2013). For example, memories for items with greater reward show selective sleep-dependent memory consolidation (Fischer and Born 2009).

However, not all studies have supported the view that sleep selectively enhances memory for salient experiences. Several studies have shown the amount of value or reward associated with items does not necessarily modulate sleep-dependent memory consolidation (Lewis et al. 2011; Tucker et al. 2011; Baran et al. 2013). Indeed, when post-sleep task performance determines how much reward can be gained, sleep can impair memory performance (Stamm et al. 2014).

While research has begun to explore how different stimuli properties affect sleep-dependent memory such studies used discrete, sequentially presented stimuli, with fixed temporal durations. This stands in contrast to real-world settings, where stimuli such as objects exist embedded in a spatially organized context and items are often encountered on several different occasions as part of travel through the world. The relative locations of places in the real world can play an important role in structuring our memories (Mou and McNamara 2002; Mou et al. 2004, 2007). Some studies have provided evidence that sleep improves spatial memory for environments encoded just prior to sleep (Ferrara et al. 2006, 2008; Wamsley et al. 2010; Coutanche et al. 2013). However, little research has explored whether spatial arrangement influences sleep-dependent consolidation of memory of object properties.

An important property of all objects is their value. Despite mixed findings on the effects of value on sleep-dependent memory consolidation, as stated above, it seems plausible that memory for the value of high-value objects would be enhanced by sleep, given that salient items appear to be more affected by sleep (Walker 2010; Perogamvros et al. 2013). Alternatively, drawing on evidence that sleep serves to generalize events (Lewis and Durrant 2011; Inostroza and Born 2013; Stickgold and Walker 2013), the reverse could be hypothesized—high-value objects might be remembered as more similar to low-value objects after sleep. This increasing similarity would be indicative of generalization. This is because it would imply that subjects were less able to retrieve the exact value of each object, and instead based their estimate on the average value of objects, generalizing across multiple experiences to obtain the average value. Here, we tested three hypotheses using a virtual simulation of an urban street network containing objects with different values: (1) sleep will lead to either a reduction or enhancement in the value of salient high-value objects, (2) spatial arrangement of the objects will mediate this effect and (3) sleep will improve memory for the layout of the objects. With regard to hypothesis 1: if generalization occurs, there should be both a reduction in the value of high-value objects and an increase in the value of low-value objects, so that all objects demonstrate a drift toward a learned mean-value of the objects.

## Materials and Methods

Sixty participants (42 females, mean age 21.9 (18–30) yr) were randomly assigned to one of the three groups: Wake, Sleep, and Immediate. Six participants were excluded due to chance level performance in our proximity test (*n* = 3 Wake, *n* = 1 Sleep, *n* = 2 Immediate). Data for 54 remaining participants (Wake *n* = 17, Sleep *n* = 20, and Immediate *n* = 17) were analyzed. None of the participants had a history of medical, neurological, or psychiatric disorders. Participants gave written informed consent and the study was approved by the University College London (UCL) ethics committee.

The study comprised two sessions of training and testing, with an 11-h retention interval for Wake group and Sleep group. Participants in the Wake group were asked to refrain from sleeping and participants in the Sleep group were encouraged to sleep at least 6 h during night. The Immediate group retention interval was 15 min.

**Figure 1.**
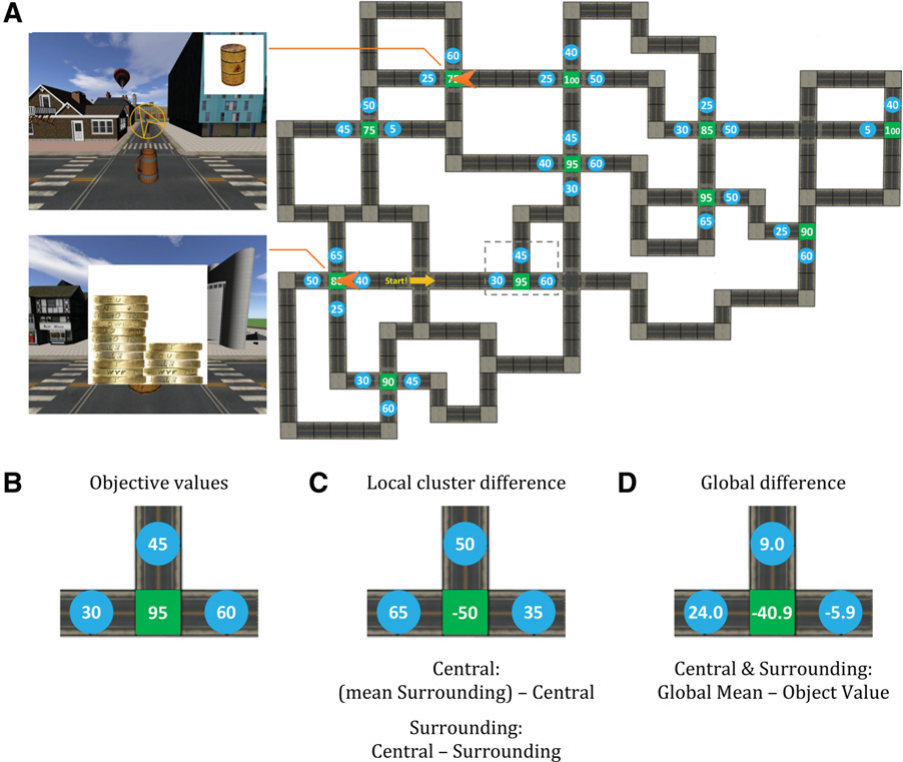
(*A*) Map of the environment and task stimuli. (*Right*) Map shown with green squares and blue circles indicating the location of “Central” and “Surrounding” objects, respectively. Numbers indicate the values of the items. The yellow arrow represents location and heading direction of the beginning of the navigation task. Orange arrows on the map show location and heading direction for two screenshots during the task (*left*). The “target object” is displayed on the *top right-*hand side of the screen. The directional yellow arrow in the view points toward the location of the target object. This arrow appeared immediately and after 5, 15, and 25 moves in blocks 1–4, respectively. It was present in all blocks, but was displayed later and later in different blocks (see Supplemental Table 1). The coins corresponding to each object appeared when the target object was found. The pile of coins was displayed for a total of 3 sec. (*B*) Close-up view of an example cluster, highlighted by dashed rectangle in the map above. (*C*) “Local cluster difference” assigned to each objects presented in (*B*). This value is calculated differently for Central and Surrounding objects. In this example, the central object local cluster difference is −50 based on (30 + 45 + 60)/3−95 and for surrounding object on the *left-*hand side the local cluster difference is 65, based on 95−30. (*D*) Represents the “global difference” of values of the objects shown in *B*. Global mean is the mean of value of all the objects (=54.05). For the central object 54.05 − 95 = −40.95 and for the surrounding object on the *left-* and *right-*hand sides 54.05 − 60 = −5.95 and 54.05 − 30 = 24.05, respectively.

The training task comprised a virtual town in which participants navigated using four keyboard arrow buttons (Vizard virtual reality software toolkit v4, www.worldviz.com). The virtual city was modeled using SketchUp (www.sketchup.com) and contained numerous different buildings and 22 street junctions (see Fig. 1A). Landmarks such as cars, lampposts, and bins provided local navigational landmarks and four distinct objects formed distal landmarks in the sky, Figure 1A.

Forty-two common objects (e.g., cup, chair) were placed on the roads, which served as target objects to be navigated to. These were selected from the 3D Warehouse (3dwarehouse.sketchup.com). Some objects were placed at the center of some junctions (“Central objects”) and other objects were placed nearby the junctions (“Surrounding objects”). Objects were all different in order to avoid confusion. Central objects and their immediate surrounding objects created “clusters” of objects. A total of 11 clusters were created, resulting in 11 Central objects and 31 Surrounding objects, Figure 1A. A value was assigned to each object. These values were presented to participants as piles of £1 coins, see Figure 1A. We displayed coins rather than numbers to reduce the degree of explicit verbal encoding of the numerical value with the object. Central objects had 15, 16, 17, 18, 19, and 20 coins. The Surrounding objects had 1, 5, 6, 7, 8, 9, 10, and 11 coins. Piles of 2, 3, and 4 coins were not used to further make explicit encoding less amenable. However, images of a single coin were used to enable subjects to identify the lowest possible value.

Because the range of coins in the images presented during training and the value ratings task occurred on different scales (1, 5–11, and 15–20 coins versus 1–10), the number of coins present in the images and the ratings were converted to the 10–100 scale with the following formulae: *value* = *(90* × #*coins* + *100)/19* and *value rating* = *10* × *rating*. In order to be able to investigate the influence of values of Central and Surrounding objects on the subjective ratings independently, value of objects were distributed in the way that there was no significant correlation between value of the Central and the mean of the Surrounding objects (*r*_(11)_ = 0.059, *P* = 0.863) and between each Surrounding object and its Central object (*r*_(31)_ = 0.013, *P* = 0.944).

The training session involved learning to navigate to objects in the virtual town. The “target object” was displayed in the top right-hand corner of the screen, Figure 1A. When objects were reached, the pile of coins representing the value of that object was displayed overlaying the object, indicating the potential reward of that object, Figure 1B. Participants were instructed that they should not be concerned with counting the number of coins on the screen, but to instead obtain an estimate of the value of each object. This session was split over four blocks. Participants were told that they would be penalized for every step during navigation in the last three blocks. They were told that they would receive monetary reward proportionate to the collected values at the end of the study. This way they were encouraged to take the optimal path to the targets. Participants were notified of their score at the end of every block. The score was presented in terms of value of the collected coins (as calculated above) and the penalties (five points per moved segment) to dissociate the points and coins. This was carried out to discourage participants from simply counting the coins.

Participants were told that after training they would be tested on their ability to maximize their reward by choosing between pairs of objects and navigating to the chosen object along an optimal path. This was to focus subjects on learning the layout of the objects and their values. After training, participants were asked to rate the value of each object on a scale and make proximity judgments about pairs of objects. We used this approach as we wanted to focus participants on acquiring an integrated knowledge of each object's location and value during training, while also obtaining, from our test phase, separate measures of the participants’ subjective estimates of each object's value and their knowledge of their spatial relationships. However, participants were not tested as they were expected to be. To obtain explicit measures of value and spatial knowledge two tasks were given. “Value Rating Task”: objects were presented alongside a scale of money, which represented a pile with one coin (representing the smallest value) at one end and a pile with 20 coins (representing the maximum value) at the other end, Supplemental Figure 3A. Keys 1–10 on the keyboard (0 key was used for 10) were used to indicate their memory for value. For the analysis, ratings were multiplied by 10 to achieve values between 10 and 100. “Proximity Test”: one Surrounding object was displayed on the left-hand side of the screen alongside a pair of Central objects on the right-hand side of the screen, one from the same cluster and one from another cluster. Participants had to select which Central object was closest to the Surrounding object, see Supplemental Figure 3B and Supplemental Material.

## Results

In order to compare performance of the participants in the Training session four parameters were compared between groups (Wake/Sleep/Immediate) using analysis of variance (ANOVA) with total duration of Training session, mean number of moves per trial, number of visits Central and Surrounding objects per object as dependent variables. These analyses showed no significant differences between any of the groups (*P*s > 0.076, see Supplemental Results;Supplemental Table 2).

To investigate whether participants acquired a reasonable knowledge of the location of the objects, three one-sample *t*-tests were conducted to compare performance in the Proximity Test against chance performance (50%). All three tests showed performance significantly differed from chance performance (*P*s < 0.001). Additionally, no differences in performance were evident between the groups (one-way ANOVA with group as independent variable and accuracy (*F*_(2,51)_ = 1.22, *P* = 0.304) and response time (*F*_(2,51)_ = 1.79, *P* = 0.117) as dependent variables), see Supplemental Results for details.

**Figure 2.**
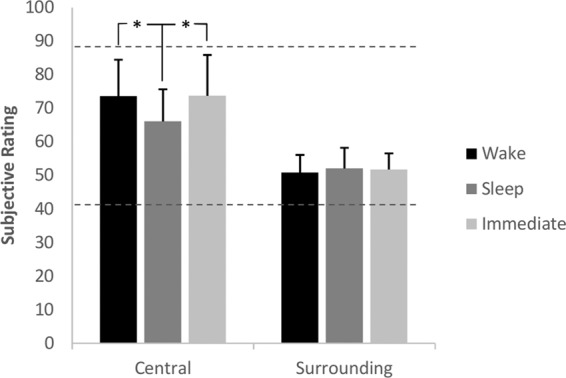
Mean subjective rating of value of the objects for all three groups. The dashed lines represent mean objective values for Central (*upper* line, 89.091) and Surrounding (*lower* line, 41.29) objects. (*) *P* < 0.05. The error bars represent 1 SD.

In the Value Rating task participants in all three groups showed significant correlations between their value ratings of the Central (*P*s < 0.01) and Surrounding (*P*s < 0.001) objects and the actual values of objects (see Supplemental Results Fig. 4; Supplemental Table 3). This shows that subjects could memorize the value of the objects. While there were no significant differences between groups on our Proximity Test, we did observe a group by object-type (Central versus Surrounding) interaction on the Value Rating Task (interaction effect in an ANOVA with group and object type as independent variable and subjective value as dependent variable *F*_(2,51)_ = 3.78, *P* = 0.029, see Supplemental Results Figure 5). For Central objects post hoc independent sample *t*-tests showed a significant difference between Sleep and Wake groups (*t*_(35)_ = 2.45, *P* = 0.019) and a significant difference between Sleep and Immediate groups (*t*_(35)_ = 2.45, *P* = 0.020) with the Sleep group underestimating the values more than Wake and Immediate groups. No other comparisons were significant. One-sample *t*-tests revealed that the value of Surrounding objects were overestimated by all groups (*P*s < 0.001) and Central objects were underestimated by all groups (*P*s < 0.001), Figure 2. Additionally paired-sample *t*-tests showed Central objects were significantly more underestimated than overestimation of Surrounding objects in the Sleep group (*t*_(19)_ = 4.30, *P* < 0.001) but not in the Wake nor Immediate groups (*P*s > 0.175).

**Figure 3.**
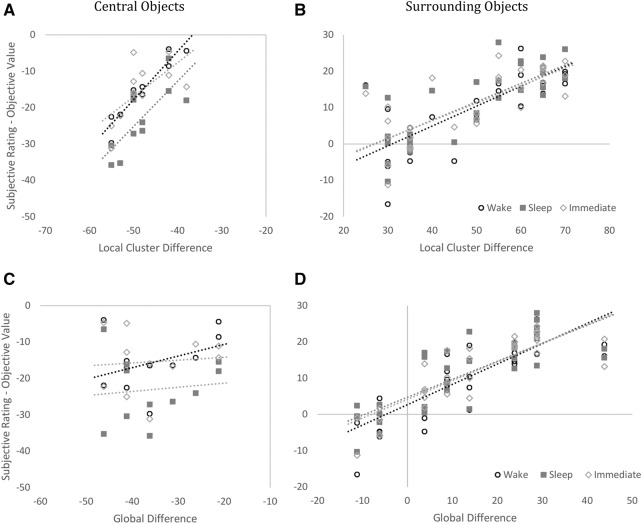
Scatter plots showing correlation of (*A*–*B*) local cluster difference and value rating error and (*C–D*) global difference and value rating error. Value rating errors for the Central objects showed a significant correlation with Local Cluster difference but not for Global Difference. Value rating errors for the Surrounding objects, however, showed a significant correlation with both Local Cluster and Global differences. Each plotted marker represents the mean subjective value rating for one of the objects. See also Supplemental Table 4.

To test for local and global effects on generalization of value we examined the correlation between subjects’ value ratings and: (a) with the error predicted by the difference in local cluster values and (b) with the error predicted by the global mean value (Fig. 1B–D). While Surrounding objects were consistent with both a shift to the global mean and a shift to the local Central object value (*P*s < 0.001), Central objects were only consistent with a shift to the local cluster mean value (*P*s < 0.001 and *P*s > 0.44 for global difference) (see Fig. 3; Supplemental Table 4). Indeed, for the Central objects for all three groups, the mean correlation between value ratings error and local cluster mean was significantly more positive than the correlation between value ratings error and the global mean value (*P*s < 0.001, see Supplemental Table 5). This implies that, while the error in ratings for the Surrounding objects can be explained by an overall less precise memory, the error for the value of Central objects was driven by specific generalization of the Central objects value to the value of the local Surrounding objects.

## Discussion

Using a novel virtual reality navigation task we find that sleep enhances the generalization of learned value specifically for high-value objects at the center of clusters. Our study revealed a number of effects. First, independent of delay or sleep, subjects showed a consistent underestimation of the value of high-value central objects and an overestimation of the value of low-value surrounding objects. Second, for central high-value objects this underestimation was explained by the influence of local surrounding objects, rather than by a shift to the global mean value. Thus, the generalization of value for high-value objects was spatially mediated, and not simply a general reduction in memory for value. Finally, the effect of sleep was specific to value representations; sleep had no impact on the precision of memory for the spatial layout of the objects.

Conflicting evidence suggests that sleep might specifically alter representations of salient high-value objects (Hu et al. 2006; Payne et al. 2008; Fischer and Born 2009; Wilhelm et al. 2011; Van Dongen et al. 2012; Stamm et al. 2014) or have no specific effect on salient objects (Lewis et al. 2011; Tucker et al. 2011; Baran et al. 2013). Our results support the view that sleep specifically affects memory for salient, motivationally relevant stimuli. This inconsistency could be explain by (1) the fact that we had a continuum of values as compared with only two conditions in previous studies (Lewis et al. 2011; Tucker et al. 2011). (2) Additionally, we did not have any “do not remember” condition as in Baran et al. (2013). In their study, participants responded as “do not know” in 30.4%. We argue that they may have failed to notice any difference in estimation of value because participants responded to value question only when they were certain. While a number of studies have found sleep-related enhancement memory for salient stimuli (Hu et al. 2006; Payne et al. 2008; Fischer and Born 2009; Javadi et al. 2011; Wilhelm et al. 2011; Van Dongen et al. 2012), in the case of spatial navigation sleep has been reported to impair memory for rewarded objects (Stamm et al. 2014). In addition, memory for items seen again in the same context has also been found to be worse after sleep (Cairney et al. 2011). Our results provide further evidence that sleep can impair memory for object attributes, specifically for salient high-value objects, consistent with the view that sleep targets salient object representations (Lewis and Durrant 2011; Inostroza and Born 2013; Stickgold and Walker 2013).

While the negative impact of sleep on memory is consistent with Stamm et al. (2014), a number of differences in experimental design between our study and Stamm et al. (2014) should be considered. Akin to our study, Stamm et al. (2014) had participants learn to navigate to objects associated with value in a virtual town. In the key condition used by Stamm et al. (2014) subjects had to travel to the objects as fast as possible to obtain the maximum reward because the reward perished over time. Thus, how to navigate to each object was learned through aversive feedback. In contrast, our participants were warned that they would be rewarded by performance, but no explicit value-related feedback was presented during learning, and thus no aversive memories (e.g., “I wish I had been faster”) were being formed during learning. Another notable difference is that Stamm et al. (2014) tested memory by having subjects navigate, rather than rating the amount of value they expect to obtain. Stamm et al. (2014) make a clear argument for the involvement of cortisol during sleep mediating their effects in relation to the stress experienced during learning prior to sleep. Due to the nonaversive nature of our task, we doubt this accounts for our effects.

If our results are not as a result of the same process as Stamm et al. (2014), what is mediating them? One strong candidate is the sleep-related generalization/abstraction of stimulus properties in memory. Current theories argue that frontal and temporal lobe brain regions extract generalizations from sensory experiences, allowing for the development of long-term semantic memory (Diekelmann and Born 2010; Lewis and Durrant 2011; Inostroza and Born 2013). The advantage of such a system is that it can generalize across sensory evidence to build a model of the world and make predictions. The downside of generalization is that fine-grained detail may be lost. In our experiment, subjects could have made errors by either: (a) a general decline in the precision of the value of each object, resulting in a random error around the mean or (b) a generalization across the other objects leading to a systematic error in recalled values. In support of generalization, our data show that value ratings for high-value central objects shifted toward a generalized value from other surrounding objects.

**Figure 4.**
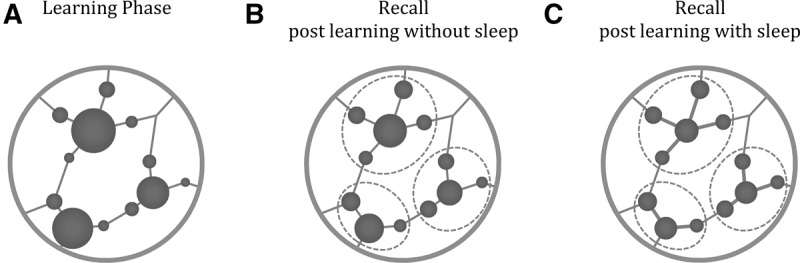
Theoretical perspective on the spatially mediated generalization. Panels show a section of the environment with objects (circles) and paths (lines). The larger the circle; the larger the value. (*A*) Initially participants learn object locations and values. Circle diameter represents the objective value of the objects. (*B*) At later recall the low-value objects are overestimated and high-value objects are underestimated, as illustrated by the changes in the diameter of the circles. (*C*) The central objects, but not surrounding objects, become even further underestimated after sleep. Future research will be useful to determine if the same effects occur if the high-value objects are located at the edge of clusters.

One problem with interpreting a shift to the mean as evidence of a generalization is that such a shift may be confounded with a tendency to opt for the middle of the scale when memory is poor. Indeed, for low-value surrounding objects we cannot rule this out. In contrast, our data provide evidence against this general shift for the high-value central objects. We found no correlation between ratings and the ratings predicted by a shift to the mean, Figure 3C. Instead, we found value ratings were predicted by a shift toward a local cluster mean. Since the local cluster mean could be distinguished from the global mean, our data imply that subjects specifically generalized the ratings from the high-value objects to their local surrounding objects. This was the case for all three groups and was significantly enhanced by sleep (see Fig. 4 for a diagram explaining how this process may occur). Our finding of a decrement in memory is consistent with data from Cairney et al. (2011) who found that sleep led to worse memory for items recalled in the same context. Currently, there are few studies that have reported a detrimental impact of sleep on memory precision. More research will be required to provide a clearer understanding of the processes involved.

In summary, we find sleep enhances a spatially mediated generalization of value for high-value objects to low-value objects. Our results support the view that sleep specifically has an impact upon motivationally relevant salient features for memory consolidation and generalization. We found no impact of sleep on spatial memory for the objects. Future studies combining spatial proximity, mapping and in situ navigation tasks will be useful to explore whether sleep has a differential effect across such tasks.

## Supplementary Material

Supplemental Material

## References

[JAVADILM038828C1] Baran B, Daniels D, Spencer RM. 2013 Sleep-dependent consolidation of value-based learning. PLoS One 8: e75326. 24130703 10.1371/journal.pone.0075326 PMC3793990

[JAVADILM038828C2] Cairney SA, Durrant SJ, Musgrove H, Lewis PA. 2011 Sleep and environmental context: interactive effects for memory. Exp Brain Res 214: 83–92. 21800251 10.1007/s00221-011-2808-7

[JAVADILM038828C3] Coutanche MN, Gianessi CA, Chanales AJ, Willison KW, Thompson-Schill SL. 2013 The role of sleep in forming a memory representation of a two-dimensional space. Hippocampus 23: 1189–1197. 23780782 10.1002/hipo.22157 PMC4131444

[JAVADILM038828C4] Diekelmann S, Born J. 2010 The memory function of sleep. Nat Rev Neurosci 11: 114–126. 20046194 10.1038/nrn2762

[JAVADILM038828C5] Ditye T, Javadi AH, Carbon C-C, Walsh V. 2013 Sleep facilitates long-term face adaptation. Proc Biol Sci 280: 20131698. 23986109 10.1098/rspb.2013.1698 PMC3768314

[JAVADILM038828C6] Durrant SJ, Taylor C, Cairney S, Lewis PA. 2011 Sleep-dependent consolidation of statistical learning. Neuropsychologia 49: 1322–1331. 21335017 10.1016/j.neuropsychologia.2011.02.015

[JAVADILM038828C7] Ellenbogen J, Hu P, Payne J, Titone D, Walker M. 2007 Human relational memory requires time and sleep. Proc Natl Acad Sci 104: 7723. 17449637 10.1073/pnas.0700094104 PMC1863467

[JAVADILM038828C8] Ferrara M, Iaria G, De Gennaro L, Guariglia C, Curcio G, Tempesta D, Bertini M. 2006 The role of sleep in the consolidation of route learning in humans: a behavioural study. Brain Res Bull 71: 4–9. 17113921 10.1016/j.brainresbull.2006.07.015

[JAVADILM038828C9] Ferrara M, Iaria G, Tempesta D, Curcio G, Moroni F, Marzano C, De Gennaro L, Pacitti C. 2008 Sleep to find your way: the role of sleep in the consolidation of memory for navigation in humans. Hippocampus 18: 844–851. 18493970 10.1002/hipo.20444

[JAVADILM038828C10] Fischer S, Born J. 2009 Anticipated reward enhances offline learning during sleep. J Exp Psychol Learn Mem Cogn 35: 1586–1593. 19857029 10.1037/a0017256

[JAVADILM038828C11] Fischer S, Hallschmid M, Elsner A, Born J. 2002 Sleep forms memory for finger skills. Proc Natl Acad Sci 99: 11987–11991. 12193650 10.1073/pnas.182178199 PMC129381

[JAVADILM038828C12] Hu P, Stylos-Allan M, Walker MP. 2006 Sleep facilitates consolidation of emotional declarative memory. Psychol Sci 17: 891–898. 17100790 10.1111/j.1467-9280.2006.01799.x

[JAVADILM038828C13] Inostroza M, Born J. 2013 Sleep for preserving and transforming episodic memory. Annu Rev Neurosci 36: 79–102. 23642099 10.1146/annurev-neuro-062012-170429

[JAVADILM038828C14] Javadi A-H, Walsh V, Lewis PA. 2011 Offline consolidation of procedural skill learning is enhanced by negative emotional content. Exp Brain Res 208: 507–517. 21120459 10.1007/s00221-010-2497-7

[JAVADILM038828C15] Lahl O, Wispel C, Willigens B, Pietrowsky R. 2008 An ultra short episode of sleep is sufficient to promote declarative memory performance. J Sleep Res 17: 3–10. 18275549 10.1111/j.1365-2869.2008.00622.x

[JAVADILM038828C16] Lau H, Alger SE, Fishbein W. 2011 Relational memory: a daytime nap facilitates the abstraction of general concepts. PLoS One 6: e27139. 22110606 10.1371/journal.pone.0027139 PMC3217953

[JAVADILM038828C17] Lewis P, Durrant S. 2011 Overlapping memory replay during sleep builds cognitive schemata. Trends Cogn Sci 15: 343–351. 21764357 10.1016/j.tics.2011.06.004

[JAVADILM038828C18] Lewis P, Cairney S, Manning L, Critchley HD. 2011 The impact of overnight consolidation upon memory for emotional and neutral encoding contexts. Neuropsychologia 49: 2619–2629. 21621549 10.1016/j.neuropsychologia.2011.05.009 PMC7614373

[JAVADILM038828C19] Marshall L, Born J. 2007 The contribution of sleep to hippocampus-dependent memory consolidation. Trends Cogn Sci 11: 442–450. 17905642 10.1016/j.tics.2007.09.001

[JAVADILM038828C20] Mou W, McNamara TP. 2002 Intrinsic frames of reference in spatial memory. J Exp Psychol Learn Mem Cogn 28: 162–170. 11827078 10.1037/0278-7393.28.1.162

[JAVADILM038828C21] Mou W, McNamara TP, Valiquette CM, Rump B. 2004 Allocentric and egocentric updating of spatial memories. J Exp Psychol Learn Mem Cogn 30: 142–157. 14736303 10.1037/0278-7393.30.1.142

[JAVADILM038828C22] Mou W, Zhao M, McNamara TP. 2007 Layout geometry in the selection of intrinsic frames of reference from multiple viewpoints. J Exp Psychol Learn Mem Cogn 33: 145–154. 17201558 10.1037/0278-7393.33.1.145

[JAVADILM038828C23] Payne JD, Stickgold R, Swanberg K, Kensinger EA. 2008 Sleep preferentially enhances memory for emotional components of scenes. Psychol Sci 19: 781–788. 18816285 10.1111/j.1467-9280.2008.02157.x PMC5846336

[JAVADILM038828C24] Payne JD, Schacter DL, Propper RE, Huang L-W, Wamsley EJ, Tucker MA, Walker MP, Stickgold R. 2009 The role of sleep in false memory formation. Neurobiol Learn Mem 92: 327–334. 19348959 10.1016/j.nlm.2009.03.007 PMC2789473

[JAVADILM038828C25] Perogamvros L, Dang-Vu TT, Desseilles M, Schwartz S. 2013 Sleep and dreaming are for important matters. Front Psychol 4: 474. 23898315 10.3389/fpsyg.2013.00474 PMC3722492

[JAVADILM038828C26] Rasch B, Buchel C, Gais S, Born J. 2007 Odor cues during slow-wave sleep prompt declarative memory consolidation. Science 315: 1426–1429. 17347444 10.1126/science.1138581

[JAVADILM038828C27] Spiers HJ, Bendor D. 2014 Enhance, delete, incept: manipulating hippocampus-dependent memories. Brain Res Bull 105: 2–7. 24397964 10.1016/j.brainresbull.2013.12.011 PMC4058530

[JAVADILM038828C28] Stamm AW, Nguyen ND, Seicol BJ, Fagan A, Oh A, Drumm M, Lundt M, Stickgold R, Wamsley EJ. 2014 Negative reinforcement impairs overnight memory consolidation. Learn Mem 21: 591–596. 25320351 10.1101/lm.035196.114 PMC4201816

[JAVADILM038828C29] Stickgold R, Walker MP. 2013 Sleep-dependent memory triage: evolving generalization through selective processing. Nat Neurosci 16: 139–145. 23354387 10.1038/nn.3303 PMC5826623

[JAVADILM038828C30] Tucker MA, Tang SX, Uzoh A, Morgan A, Stickgold R. 2011 To sleep, to strive, or both: how best to optimize memory. PLoS One 6: e21737. 21799746 10.1371/journal.pone.0021737 PMC3140493

[JAVADILM038828C31] Van Dongen EV, Thielen J-W, Takashima A, Barth M, Fernández G. 2012 Sleep supports selective retention of associative memories based on relevance for future utilization. PLoS One 7: e43426. 22916259 10.1371/journal.pone.0043426 PMC3420871

[JAVADILM038828C32] Walker M. 2010 Sleep, memory and emotion. Prog Brain Res 185: 49–68. 21075233 10.1016/B978-0-444-53702-7.00004-X

[JAVADILM038828C33] Walker M, Stickgold R. 2010 Overnight alchemy: sleep-dependent memory evolution. Nat Rev Neurosci 11: 218. 20168316 10.1038/nrn2762-c1 PMC2891532

[JAVADILM038828C34] Wamsley E, Tucker M, Payne JD, Stickgold R. 2010 A brief nap is beneficial for human route-learning: the role of navigation experience and EEG spectral power. Learn Mem 17: 332–336. 20581255 10.1101/lm.1828310 PMC2904102

[JAVADILM038828C35] Wilhelm I, Diekelmann S, Molzow I, Ayoub A, Mölle M, Born J. 2011 Sleep selectively enhances memory expected to be of future relevance. J Neurosci 31: 1563–1569. 21289163 10.1523/JNEUROSCI.3575-10.2011 PMC6623736

