# Supplementary material for: Sleep enhances a spatially mediated generalization of learned values
Source: Learn Mem. 2015 Oct;22(10):532–6. doi: 10.1101/lm.038828.115 (PMC4579355; doi:10.1101/lm.038828.115)
Supplement: Supplemental Material [file supp_22_10_532__index.html]

Supplemental Material 

# Sleep enhances a spatially mediated generalization of learned values

## Supplemental Material

**Files in this Data Supplement:**

- Supp Material.pdf
