# Supplementary material for: Sleep enhances a spatially mediated generalization of learned values
Source: Learn Mem. 2015 Oct;22(10):532–6. doi: 10.1101/lm.038828.115 (PMC4579355; doi:10.1101/lm.038828.115)
Supplement: Supplemental Material [file supp_22.10.532_SuppMaterial.pdf]

Sleep enhances a spatially-mediated generalisation of learned values

Amir-Homayoun Javadi, Anisha Tolat, Hugo J. Spiers

1 Extended Method

1.1 Apparatus and Stimuli

Apparatus; PC computers with 17" LCD monitors were used throughout the task. Participants responded using regular keyboards.

Environment; Distal cues (airplane, helicopter, blimp and balloon) were placed just beyond the edge of each side of the town to help participants to orient in the map. Thus, only one of the distal cues was visible at every heading direction. Each button press executed travel in the direction specified by that button (e.g. 'Left') to the next junction in the environment. Within the virtual environment, some objects were placed at the centre of some junctions (*Central objects*) and other objects were placed at a distance (20 ± 5 metres) around the junctions (*Surrounding objects*). Central objects and their immediate surrounding objects created *clusters* of objects.

Coin Piles; Three different images were used to display each pile of coins; e.g. in one all coins on top of each other, in another they might be arranged in a row, Figure 1. This served to make the learning of the value harder and less amenable to storing a 'visual snap shot' of the object with a number next to it.

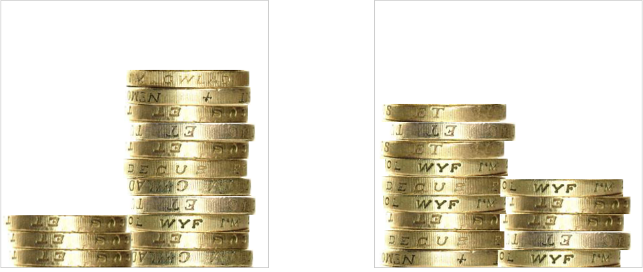

Figure 1. Sample of two piles of coins representing 14 coins each.

1.2 Procedure and Task

All participants were asked to have at least 6 hours night sleep the night before the experiment. Participants were asked to avoid any strenuous exercise, heavily smoke, take recreational

drugs, alcohol or caffeine in between the two sessions and within the 12 hours before the start of the training session. All participants participated in two sessions: Testing and Training. Three groups of participants were tested: Wake, Sleep and Immediate. We were interested in the effects of sleep and wakefulness within an 11-hour retention interval. An Immediate group with only a 15 minutes retention interval was run to establish a measurement of participants' performance shortly after training. This measurement was considered as a baseline enabling us to investigate direction and magnitude of effects of an 11-hour retention interval. Figure 2 shows the timing of the sessions for all three groups.

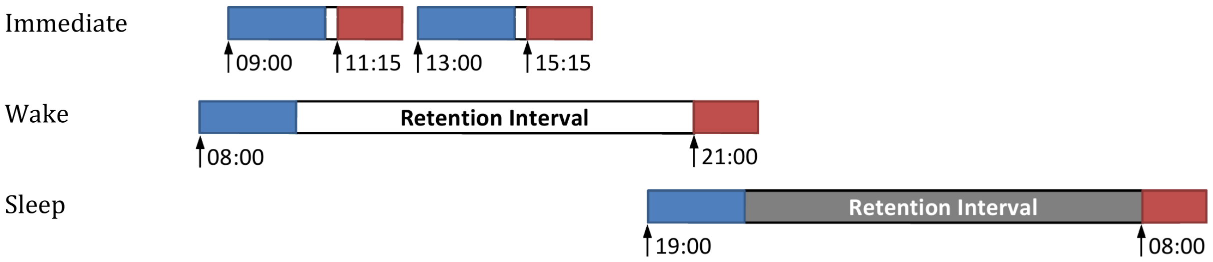

Figure 2. Experimental timeline for *Immediate*, *Wake* and *Sleep* groups. Blue and red rectangles represent *Training* and *Testing* sessions, respectively. The Training session and Testing sessions lasted 2 and 1 hours, respectively. Participants in the Wake group were trained in the morning and tested in the same evening. They spent the day as they pleased, but were required to avoid sleeping or taking part in any strenuous physical activity. Participants in the Sleep group were trained in the evening and tested in the next morning. They travelled home and slept overnight – a minimum of 6 hours was requested. Retention interval for wake and sleep groups was 11 hours. Retention interval for Immediate group was 15 minutes. During this short period participants watched a documentary on animal life, and then completed the testing session.

Training session; within the training session, participants were shown an object and were asked to navigate through the environment to find it. The participant was then asked to navigate to the next object. The training session was divided into four blocks. In each block every object was selected as the target object once. In order to help the participants to navigate to the objects a yellow arrow was displayed on the screen that portrayed the direction of the target object. This arrow was displayed later and later throughout the consecutive blocks (see Figure 1A in the main manuscript).

In blocks 1 and 2 participants were provided a paper map outlining the layout of the environment with some of the landmark buildings displayed. They were instructed to use the map by noting down every object whenever they located them. Table 1 displays a summary of the blocks of the training session.

In blocks 2, 3 and 4, participants were required to carry out the same procedure – locate every object and pay attention to the value of each one. Furthermore, they were told that for every move, 5 points deduces from their total point score. Therefore, they were encouraged to try and take the optimal path to the target object, in order to minimise their loss and hence maximise their reward.

Block	Yellow Direction Arrow	Penalty	Paper Map
1	immediately		×
2	after 5 moves	×	×
3	after 15 moves	×	
4	after 25 moves	×	

Table 1. Summary of the four blocks of the training session, which was provided to participants in the instruction manual. After the second block, participants were allowed a ten-minute refreshment break to relax and rest from viewing the screen.

A few parameters were considered in the design of the task:

1. placement of the valued objects within a spatial context
2. inclusion of as many objects as possible to have a reasonable number of samples, and
3. a sufficient distance between clusters of objects was required to form distinct clusters.

This led us to have a large environment with 42 objects. Additionally we avoided having two objects on the same street section connecting junctions. This was done to create clusters around junctions, and not street sections.

Within the information sheet given at the beginning of the training session, participants were informed that in the testing session they would have to choose between pairs of objects to navigate to, recalling which object had the highest value. Therefore, they were encouraged to always pick the object that results to a higher reward, a trade-off between value and distance. This was done to encourage subjects to memorise locations and values, in order to be able to successfully maximise their financial gain. Participants were told that they would be monetarily rewarded based on their performance in both sessions. However, this was different to the actual content of the testing session.

Testing session; within the actual testing session, participants were asked to answer questions within 2 phases. In the first phase (*Value Rating Task*), participants were shown an object on the screen and a scale of money. The scale represented the lowest value i.e. the smallest pile of coins at one end and the highest value i.e. the maximum amount of coins at the other end, Figure 3A.

Participants were asked to indicate how much value was associated to that object. Participants had to use the keys 1-10 on the keyboard (for 10, participants were instructed to use the 0 key) to indicate their feeling of the value. This phase consisted of 126 trials within 3 blocks; i.e. each object was questioned 3 times.

In the second phase of the testing session (*Proximity Test*), participants were shown one Surrounding object on the left-hand side of the screen alongside a pair of Central objects on the right-hand side of the screen, one from the same cluster and one from another one. Participants were instructed to choose which object, out of the pair, was spatially closest to the object on the left-hand side, Figure 3B. This phase consisted of one block of 93 trials; i.e. each surrounding object was tested 3 times. The same pair of Central objects was presented every time for each Surrounding object.

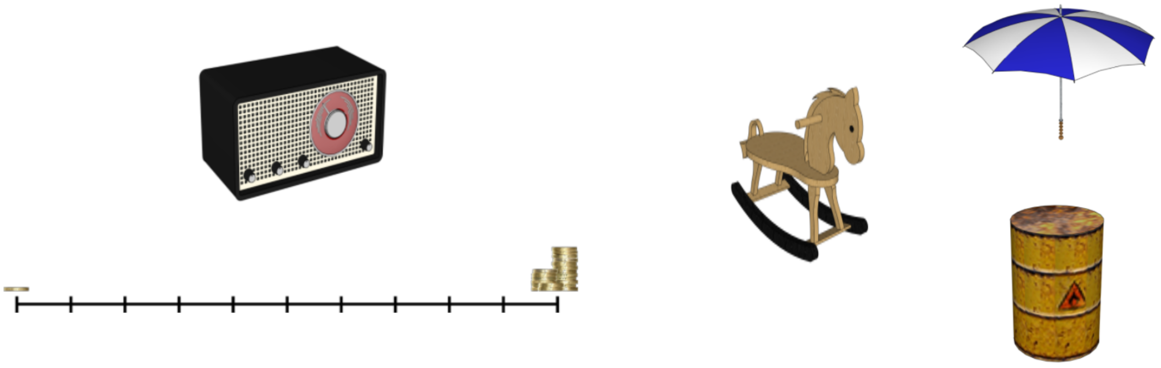

(A) (B)

Figure 3. (A) Example of a trial in phase 1 of Testing session (*Value Rating Task*). Participants were asked to rate what they felt the value of that particular object was. (B) Example of a trial in phase 2 of Testing session (*Proximity Test*). Participants were asked to pick out of the two objects on the right, the one which was spatially closest to the object on the left. All the objects presented on the left hand side were Surrounding objects and all the objects presented on the right hand side were Central objects.

At the end of the testing session participants were asked to fill in four questionnaires. They were asked to complete a standard handedness questionnaire, to assess whether they had right or left handed tendencies (Oldfield, 1971). Additionally, they were assessed on whether they were ‘morning’ or ‘evening’ people by a standard morningness-eveningness questionnaire (Horne & Ostberg, 1976). Furthermore, they were assessed on navigational skill by the Santa Barbara Scale (Hegarty, Richardson, Montello, Lovelace, & Subbiah, 2002). The log-sheet asked participants about the quality and quantity of their sleep, their activities in between the two sessions and their activities within the 12 hours before the experiment as well as their

consumption of caffeine, alcohol, recreational drugs and smoking and if they had undertaken any physical strenuous activity in order to ensure that rules were followed.

1.3 Statistical Analysis

Data handling was done using MATLAB (v2013b; MathWorks Company, Natick, MA, USA). Statistical data analysis was performed using SPSS (v22.0; LEAD Technologies Inc., Charlotte, NC, USA).

Training Session; To make sure that participants in different groups were trained similarly two one-way analysis of variances (ANOVA) were run with group (Wake/Sleep/Immediate) as the between-subject factor on the total duration of training and total number of moves.

Proximity Test; In order to analyse the participants' spatial knowledge, two one-way ANOVAs were run with group as the between-subject factor on participants' performance and response time in the Proximity Test.

Value Rating Task; Ratings were subjected to a mixed-factor ANOVA with object type (Surrounding/Central) as within subject factor and group as between subject factor. *Post-hoc* two-independent sample *t*-tests were conducted to compare ratings for different groups. Subsequently, ratings for the three groups were compared with mean of objective values for Surrounding and Central objects, 41.29 and 89.09, respectively, using one-sample *t*-test to investigate whether participants' rating had significant under- or over-estimation.

The objective value of each object was subtracted from the subjective rating to obtain a *rating accuracy score*, which was compared to the object values to determine how accurately subjects learned the values of the objects. We further analysed the data to investigate whether participants' value ratings were biased due to the value of neighbouring objects or biased relative the global mean of all objects.

The difference between the value of each Surrounding object and its immediate Central object provided a means of the *local cluster difference* for Surrounding objects. The difference between the value of each Central object and the mean value of its immediate Surrounding object provided a means of the *local cluster difference* for Central objects. The difference between the objective value of each object and the mean objective value of all objects was calculated to obtain a *global mean difference* (see Figure 1B-D in the main manuscript).

Spearman's correlation analyses were conducted on rating accuracies and difference between centre and surround values for different groups and object types. A Bonferroni correction for multiple comparisons was used to account for conducting tests over three groups (i.e. we used a corrected threshold of $p < 0.016$). *Questionnaires;* Responses to the questionnaires were also

analysed to investigate possible differences between groups. One-way ANOVA was run with group as the between-subject factor on morningness-eveningness, handedness and self-rated navigation ability scores.

2 Extended Results

2.1 Training Session

One-way ANOVAs showed no significant effect of group for any of the measures recorded from the Training session (see Table 2). In order to investigate the data further, we compared the performance of the Wake and Sleep group using independent sample *t*-tests (see Table 2).

	Group			ANOVA			*t*-test	
	Wake	Sleep	Immediate	$F(2, 51)$	p	η_p^2	$t(35)$	p
Total duration (minutes)	117.31 (24.3)	126.89 (21.85)	115.98 (24.23)	1.210	0.307	0.045	1.26	0.215
Mean moves per trial	11.20 (2.58)	11.47 (2.75)	9.68 (2.85)	2.192	0.122	0.079	0.306	0.761
Number of visits to Central objects per object	54.93 (10.67)	57.60 (12.13)	48.52 (13.14)	2.706	0.076	0.096	0.704	0.485
Number of visits to Surrounding objects per object	24.00 (5.52)	25.36 (6.11)	21.24 (6.65)	2.134	0.129	0.077	0.705	0.485

Table 2. Performance in the training session. 'Moves' refers to the number of button presses used to navigate to the goal. ANOVA refers to a one-way ANOVA with group as independent factor. Values in the parentheses indicate standard deviation. *t*-test refers to comparison between Wake and Sleep groups.

2.2 Testing Session

Proximity Test

Three one-sample *t*-tests showed that participants in all three groups performed this test significant better than chance (Wake Mean % (SD) = 71.03 (18.22), $t(16) = 4.75$, $p < 0.001$, Sleep = 74.99 (14.47), $t(19) = 7.72$, $p < 0.001$, Immediate = 79.81 (16.75), $t(16) = 7.33$, $p < 0.001$). Additionally performance in between groups were compared. A One-way ANOVA with group (Wake/Sleep/Immediate) and participant's performance showed a non-significant main effect of group ($F(2, 51) = 1.22$, $p = 0.304$, $\eta_p^2 = 0.046$). Comparison of performance between Wake and

Sleep groups showed no significant different either ($t(35) = 0.73$, $p = 0.466$). Similar one-way ANOVA on response time showed a non-significant main effect of group (Wake = 2.31 (0.60), Sleep = 2.76 (0.83), Immediate = 2.45 (0.79), $F(2, 51) = 1.79$, $p = 0.117$, $\eta_p^2 = 0.066$). Similarly, comparison of response time between Wake and Sleep groups showed no significant different either (t(35) = 1.85, p = 0.071).

Value Rating Task

All groups showed evidence of learning the value of the different objects. Each group showed a significant correlation between subjective ratings of values and the objective value, for Central objects and Surrounding objects (See Figure 4 and Table 3).

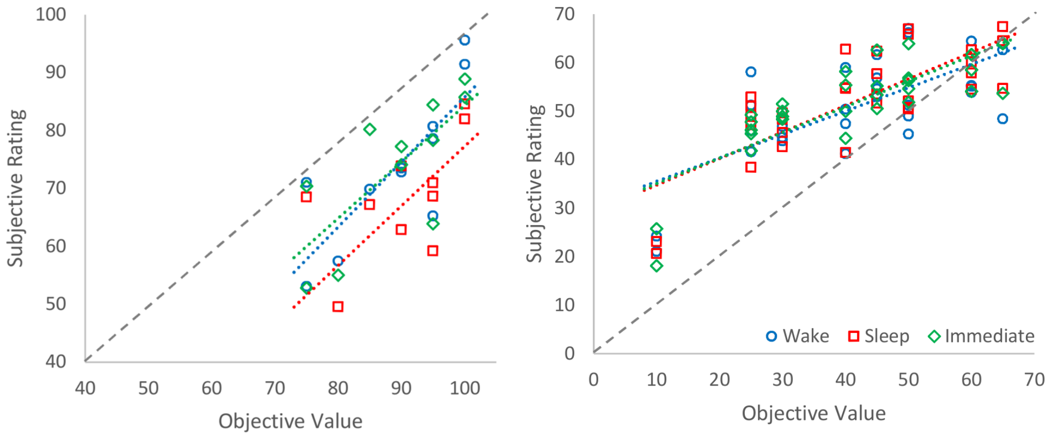

(a) Central Objects (b) Surrounding Objects

Figure 4. Scatter plots showing correlation of objective value and subjective rating. Each plotted marker represents the mean subjective rating for one of the objects.

Group	Central Objects	Surrounding Objects
Wake	$r(11) = 0.804$, $p = 0.003^*$	$r(31) = 0.632$, $p < 0.001^*$
Sleep	$r(11) = 0.707$, $p = 0.015^*$	$r(31) = 0.738$, $p < 0.001^*$
Immediate	$r(11) = 0.762$, $p = 0.006^*$	$r(31) = 0.835$, $p < 0.001^*$

Table 3. Objective values and subjective rating; * $p < 0.016$.

Differences between groups for some high value central objects were evident for the value ratings task. Mixed-factor ANOVA with object type (Surrounding/Central) and group on subjective ratings revealed a significant main effect of object type ($F(1, 51) = 174.07$, $p < 0.001$, $\eta_p^2 = 0.773$), a non-significant main effect of group ($F(2, 51) = 2.50$, $p = 0.092$, $\eta_p^2 = 0.089$) and a significant interaction of the two factors ($F(2, 51) = 3.78$, $p = 0.029$, $\eta_p^2 = 0.129$). For Central objects *Post-hoc* independent sample *t*-tests showed a significant difference between Sleep and

Wake groups ($t(35) = 2.45$, $p = 0.019$) and a significant difference between Sleep and Immediate groups ($t(35) = 2.45$, $p = 0.020$). No other comparisons were significant.

A shift in subjective ratings towards a mean value could be due to a forgetting process, whereby objects values are only recalled a coarse manner. This coarse representation would lead objects being treated more similarly to each other than they should be, which would lead to ratings being closer to the mean for all objects. According to this view, our data could be considered as evidence that sleep results in less precise memory for value for high value objects. An alternative possibility is that the spatial clustering of the environment leads to change in the subject's representation of value, such that each object shows a specific shift to the local mean value predicted by the difference between the value of the central and surrounding objects.

To test for local and global effects on generalisation of value we examined the correlation between the error in value ratings made by the subjects a) with the error predicted by the difference in local cluster values and b) with the error predicted by the global mean value (Table 4, see also Figure 3 in the main manuscript). Our analysis revealed that, while Surrounding objects were consistent with both a shift to the global mean and shift to the local Central object value, Central objects were only consistent with a shift to the local cluster mean value. Indeed for the Central objects for all groups, the mean correlation between ratings error nd local cluster mean was significantly more positive than the correlation between ratings error and the global mean value (see Table 5). This implies that, while the error in ratings for the surround objects can equally be explained by a generalisation over all objects and therefore potentially a generally less precise memory, the error for the Central objects, by contrast, was driven by specific generalisation of the central objects value to the value of the local Surrounding objects.

Objects	Group	Local Cluster Difference	Global Difference
Central	Wake	$r(11) = 0.956$, $p < 0.001^*$	$r(11) = 0.259$, $p = 0.442$
	Sleep	$r(11) = 0.799$, $p = 0.003^*$	$r(11) = 0.097$, $p = 0.777$
	Immediate	$r(11) = 0.725$, $p = 0.012^*$	$r(11) = 0.037$, $p = 0.914$
Surrounding	Wake	$r(31) = 0.735$, $p < 0.001^*$	$r(31) = 0.797$, $p < 0.001^*$
	Sleep	$r(31) = 0.733$, $p < 0.001^*$	$r(31) = 0.777$, $p < 0.001^*$
	Immediate	$r(31) = 0.753$, $p < 0.001^*$	$r(31) = 0.876$, $p < 0.001^*$

Table 4. Summary of Spearman's Rho correlation analyses local cluster differences and rating error and global differences and rating error; * $p < 0.016$.

		Mean (SD) correlation of rating errors		*t*-test
Objects	Group	Local Cluster Difference	Global Difference	Local Cluster Difference vs Global Different
Central	Wake	0.517 (0.249)	0.150 (0.283)	$t(16) = 5.538, p < 0.001^{*}$
	Sleep	0.416 (0.304)	0.003 (0.272)	$t(19) = 8.900, p < 0.001^{*}$
	Immediate	0.338 (0.298)	0.016 (0.326)	$t(16) = 4.420, p < 0.001^{*}$
Surrounding	Wake	0.426 (0.183)	0.488 (0.217)	$t(16) = 3.267, p = 0.005^{*}$
	Sleep	0.391 (0.225)	0.438 (0.229)	$t(19) = 2.446, p = 0.024$
	Immediate	0.382 (0.214)	0.434 (0.218)	$t(16) = 1.865, p = 0.081$

Table 5. Comparison between mean correlation of rating errors for local cluster difference and mean correlations of rating errors and global difference; * $p < 0.016$.

Log-sheets and Questionnaires; log-sheets indicated that all participants in the sleep group had at least 6 hours of sleep and participants in the wake group did not sleep in between the training and testing sessions. Questionnaires indicated that there was no significant difference between all groups for morningness-eveningness, handedness and navigational skill scores. One-way ANOVA with group as independent factor was not significant for handedness ($F(2, 51) = 0.640$, $p = 0.531$), morningness-eveningness ($F(2, 51) = 0.752$, $p = 0.476$) and navigational skill scores ($F(2, 51) = 0.907$, $p = 0.410$).

3 References

Hegarty, M., Richardson, A. E., Montello, D. R., Lovelace, K., & Subbiah, I. (2002). Development of a self-report measure of environmental spatial ability. *Intelligence, 30*(5), 425-447.

Horne, J., & Ostberg, O. (1976). A self-assessment questionnaire to determine morningness-eveningness in human circadian rhythms. *International Journal of Chronobiology, 4*(2), 97-110.

Oldfield, R. (1971). The assessment and analysis of handedness: the Edinburgh inventory. *Neuropsychologia, 9*(1), 97-113.
